# Combined Effects of NMES and Mendelsohn Maneuver on the Swallowing Function and Swallowing–Quality of Life of Patients with Stroke-Induced Sub-Acute Swallowing Disorders

**DOI:** 10.3390/biomedicines8010012

**Published:** 2020-01-12

**Authors:** Haewon Byeon

**Affiliations:** Department of Speech Language Pathology, School of Public Health, Honam University, 417, Eodeung-daero, Gwangsan-gu, Gwangju 62399, Korea; bhwpuma@naver.com; Tel.: +82-10-7404-6969

**Keywords:** compound intervention program, stroke, functional dysphagia scale, Mendelsohn maneuver, neuro muscular electrical stimulation, swallowing–quality of life

## Abstract

It is necessary to identify how to improve the swallowing-related quality of life, as well as the swallowing function, in order to evaluate the effect of treatments on swallowing disorders. This study aimed to prove the effects of a compound swallowing intervention (Mendelsohn maneuver + neuromuscular electrical stimulation (NMES)) on the swallowing function and the quality of life by applying the compound swallowing intervention to patients with sub-acute swallowing disorders due to cerebral infarction for eight weeks. This study analyzed 43 subjects who were diagnosed with swallowing disorders due to cerebral infarction. The experiment consisted of the Mendelsohn maneuver treatment group (*n* = 15), the NMES treatment group (*n* = 13), the compound intervention group (Mendelsohn maneuver + NMES; *n* = 15). The results of ANCOVA showed that the changes in Functional Dysphagia Scale (FDS) scores and Swallowing–Quality of Life (SWAL–QOL) score were different among groups. The compound intervention group had the highest FDS scores and SWAL–QOL score followed by Mendelsohn, and the NMES group had the lowest. The result of this study suggests that NMES can be more effective when it is combined with a traditional swallowing rehabilitation therapy rather than a single intervention method.

## 1. Introduction

Stroke is very like to have sequelae, even if surgical treatment is successfully conducted and the patient survives. Among various sequelae, the onset rate of swallowing disorders is the highest. Previous studies have reported that the onset rate of swallowing disorders in patients with acute cerebral infarction varies greatly, ranging from 37% to 65% [1,2]. Particularly, special attention should be given to patients with acute cerebral infarction, because they are highly likely to experience aspiration, which means that food passes through the airway. Approximately 20% of patients with cerebral infarction die from aspiration pneumonia within a year from the onset; it was also reported that one in three patients with sub-acute cerebral infarction and aspiration had silent aspiration, which showed no observable symptom [3]. Therefore, active swallowing rehabilitation is needed from the onset of a swallowing disorder, to maintain the life of the patient.

On the other hand, the difficulty in swallowing not only causes medical problems but also lowers the quality of life, because swallowing is one of the most fundamental demand of human beings and a complex activity for maintaining social relationships with others. Patients with swallowing disorders experience fear and anger due to aspiration during mealtimes and consequently avoid eating with others, eventually feeling social isolation [4,5]. Moreover, Critchlow et al. [6] showed that extended intubation feeding resulted in a loss of appetite, along with depression. Therefore, it is necessary to identify how to improve the swallowing–quality of life, as well as the swallowing function, in order to evaluate the effect of treatments on swallowing disorders.

Traditional treatments to improve the swallowing function include compensatory strategies, such as posture change and maneuver and rehabilitation techniques, that strengthen the muscles associated with swallowing by exercising them repetitively [7,8]. Among these various treatments, the Mendelsohn maneuver, which focuses on submandibular hyolaryngeal muscles, has been used in clinical practice to effectively improve the function of muscles associated with laryngeal elevation [9]. The Mendelsohn maneuver is a method designed to increase the willing movement of the larynx and hyoid bone while pharyngeal swallowing is progressing [9,10]. Moreover, it is a method to voluntarily hold the position after contacting the larynx and raising it to the maximum height, and maintain it for several seconds [9,10]. It has been reported that it is effective in recovering the swallowing of patients with swallowing disorders in the pharyngeal stage [9,10].

Recently, neuromuscular electrical stimulation (NMES) has been conducted for rehabilitating the swallowing in the pharyngeal stage, and it has been continuously reported that it has a significant effect on the recovery of the swallowing function [11,12]. Many studies have consistently shown that NMES is effective for compensatory strategies and rehabilitation therapy [11,12]. On the contrary, several studies reported that NMES was not effective [13,14,15,16,17]. Therefore, further studies are needed to verify the effectiveness of NMES. Above all, most of the previous studies compared the treatment effects of NMES and those of an individual traditional therapy, and only a few studies (e.g., Li et al. [14]) have examined the effectiveness of compound interventions, including Li et al. [14].

The previous studies comparing the treatment effects of NMES and those of an individual traditional therapy have the following limitations. First, most of them simply confirmed the improvement of the swallowing function owing to an individual intervention. Although it is very rare to conduct an individual intervention for treating a swallowing disorder in the clinical practice, there is not enough evidence for the application of complex swallowing treatment programs to patients. It is very difficult to apply the results of studies that evaluated the effects of an individual treatment on the swallowing disorder to clinical practices as it is. Second, studies that did not find the effectiveness of NMES [18,19] compared NMES with traditional swallowing treatments. However, they are limited in verifying the effectiveness of NMES because they had a small sample size, measured only twice (pre-treatment and after-treatment), and analyzed short-term treatment effects within 4 weeks. Therefore, longitudinal studies are needed to verify the effectiveness by measuring changes over a sufficient period that neurological changes (recovery) owing to a treatment can be expected. Third, most studies analyzed the changes by comparing only physiological indicators related to the swallowing functions, such as aspiration, with the control group. Since swallowing has a very complex mechanism, it is impossible to identify the overall recovery of swallowing just by using physical indicators. Therefore, further verification efforts should be carried out by using various indicators, such as the quality-of-life, to prove the effectiveness of swallowing therapies. Fourth, although the ultimate goal of rehabilitation therapies is to recover functions and improve the quality of life through goal-oriented and comprehensive intervention, only a few studies evaluated the swallowing function and the quality of life at the same time.

This study aimed to (1) prove the effects of a compound swallowing intervention (Mendelsohn maneuver + NMES) on the swallowing function and the quality of life by applying the compound swallowing intervention to patients with sub-acute swallowing disorders due to cerebral infarction for eight weeks and (2) provide a basis for applying it to the rehabilitation clinics by comparing the effects of the compound swallowing intervention and an individual swallowing treatment.

## 2. Methods and Materials

### 2.1. Study Subjects

**Figure 1:**
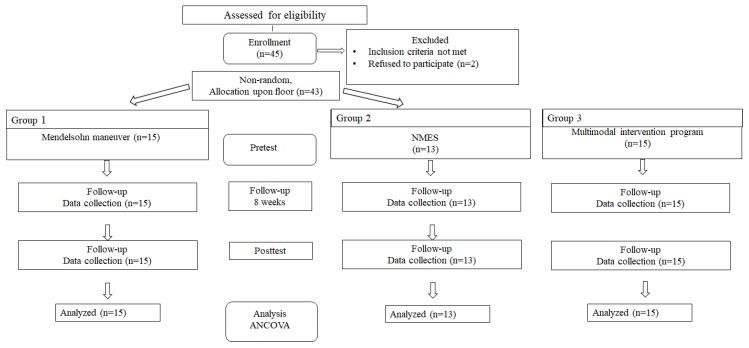
The schematic diagram of the study.

The study was designed using a nonequivalent control group pretest–posttest design. This study used 55 subjects who were diagnosed with swallowing disorders due to cerebral infarction at rehabilitation departments of four general hospitals located in Seoul and Incheon between July 2018 and January 2019 and agreed to participate after understanding the contents of this study. This study was approved by the Institutional Review Board of Honam University (Date: 2018. 12. 12, #IRB: 1041223-201812-HR-26) and was conducted in accordance with the ethical standards of the Declaration of Helsinki. The appropriate sample size was calculated using the effect-size classification method of Cohen (2003) [20]. The results of the power analysis showed that the study would require 13 subjects per group for three groups when it is a two-tailed test, alpha = 0.05, power = 0.8, medium level effect size = 0.4, and five repeated measures. However, this study recruited 45 subjects (15 subjects per group), considering potential dropouts and two subjects dropped out of the study. Therefore, this study analyzed 43 subjects. The experiment consisted of the Mendelsohn maneuver treatment group (*n* = 15), the NMES treatment group (*n* = 13), and the compound intervention group (Mendelsohn maneuver + NMES; *n* = 15) (Figure 1). The selected subjects met the following five criteria. First, they were 60 years or older and were diagnosed with swallowing disorders due to cerebral infarction. Second, they were diagnosed with swallowing disorders within the past six months. Third, they received 20 points or more in the Korean Mini-Mental State Examination (K-MMSE) [21], and they had no difficulty in understanding and conducting the test method. Fourth, they never received any swallowing treatment before participating in this study. Fifth, they agreed to participate in this study. The characteristics of the subjects are presented in Table 1.

### 2.2. Measurements

The age, gender ratio, mean monthly household income, the highest level of education, longest occupation, VFS, and the homogeneity of time since dysphagia of three groups (the Mendelsohn intervention group, the NMES group, and the compound intervention group) were tested by using one-way ANOVA and chi-square analysis. The results showed that there were no significant differences among these groups for any of these variables (Table 1).

### 2.3. Measurement

#### 2.3.1. Mendelsohn Maneuver

The Mendelsohn maneuver was performed in seven steps, with 30 min per session. First, a small amount of food or drink was put in the mouth of a subject. When the subject had a risk of aspiration, dry swallowing was prepared. Second, the thyroid cartilage was grabbed with the thumb and the index finger. Third, the therapist instructed the subject to conduct dry swallowing. Fourth, the patient pulled the thyroid cartilage upward while swallowing. Fifth, the subject should maintain the highest mobile position of the thyroid cartilage for 2 s. Sixth, the subject should feel relaxation. Seventh, the subject repeated steps 1 through 6 for 15–20 times.

#### 2.3.2. NMES

For the NMES group, swallowing rehabilitation was conducted by using VitalStim (Chattanooga Group, Hixson, TN, USA), which is a two-channel electric stimulator approved by the Ministry of Food and Drug Safety. The frequency of vibration, the stimulus width, and the stimulus per cycle were set to 80 Hz, 300 usec, and 700 usec, respectively. The lowest intensity of the current was 6.5 mA, and it was increased by 0.5 mA within the range that a subject could stand the discomfort and pain. The stimulus was given for 30 min per session. The instrument used two circular-shaped pads (diameter = 2 cm). The two electrodes were attached to the superior to the hyoid bone, and the superior to the thyroid notch [15].

#### 2.3.3. Compound Intervention Program

For the compound intervention application group, Mendelsohn maneuver intervention was applied for 15 min and NMES intervention was applied additionally for 15 min. For all treatment groups, the program was introduced and the baseline was measured in the first session. A 30-min intervention session was given to a subject from the second session to the 16th session.

#### 2.3.4. Swallowing Function

The effects of a treatment on the recovery of swallowing were evaluated by using the Functional Dysphagia Scale (FDS) [22], which was conducted by a physiatrist who was not involved in this study. For VFSS, this study used Multistar TOP (Siemens, Erlangen, Germany) and analyzed video data using virtualdub v1.10.2 (virtualdub, Seoul, Korea), which is capable of analyzing 30 frames per second.

FDS is an index for evaluating overall swallowing issues such as aspiration and the amount of residual food that are observed in the videofluoroscopic swallowing study. The maximum score is 100 and a higher score means more serious swallowing issues. The test items are composed of lip closure, bolus formation, residue in the oral cavity, oral transit time, triggering of pharyngeal, swallow fluid, laryngeal elevation and epiglottic closure, residue in valleculae, residue in pyriform sinuses, coating of the pharyngeal wall after swallow fluid, and pharyngeal transit time. When FDS test was developed, the sensitivity of the test was 81% and the specificity of the test was 70.7% [22].

#### 2.3.5. Swallowing-Related Quality of Life

Swallowing–quality of life (SWAL–QOL) [23] was used to evaluate the effects of swallowing on the quality of life. SWAL–QOL is a self-reported testing tool that consists of 44 items in 11 domains (i.e., burden, food selection, eating duration, eating desire, fear, symptoms and frequency, sleep, fatigue, communication, mental health, and social). Each item is measured by a 5-point scale (1 point: ‘very agree; 2 points: ‘agree’; 3 points: ‘not agree or disagree’; 4 points: ‘disagree’; and 5 points: ‘very disagree’). The total score of SWAL–QOL ranges from 44 to 220. A higher score indicates a higher quality of life. At the time of development, the reliability of SWAL–QOL was 0.85 and the validity of it was 0.95 [23].

#### 2.3.6. Longest Occupation

The longest occupation was determined based on the answer to the question of “What was the job you worked for the longest in your life?” The surveyed occupations can be compared with occupations internationally. This study added unemployed people, homemakers, and students, which are part of the economically inactive population, to the major classification of the 6th Korean Standard Occupation Classification [24]. As a result, this study classified subjects into manual workers (e.g., skilled workers, farmers, foresters, and fishermen), non-manual workers (e.g., clerks), and economically inactive population (e.g., homemakers and unemployed workers).

### 2.4. Statistical Analyses

This study used a nonequivalent control group pretest–posttest design. This study measured FDS and SWAL–QOL before treatment application (baseline) and after the intervention (after 8 weeks) over the total 8 weeks of the experiment period. ANCOVA was used to determine the significant differences between pre- and post-outcome variables among the three groups. We originally aimed to conduct repeated measures ANOVA in order to identify the effects of the intervention on the three groups after the intervention. However, because the study had a small sample size, and both the pretest score and the posttest score were autocorrelated, ANCOVA was used to analyze the changes in FDS and SWAL–QOL after the treatment by using the pretest as covariates. Statistical difference was determined at alpha = 0.05. All statistical analyses were conducted by using IBM SPSS version 24.0 (IBM Inc., Chicago, IL, USA).

## 3. Results

### 3.1. Pre-Homogeneity Test for FDS and SWAL–QOL

FDS (Table 2) and SWAL–QOL (Table 3) of the Mendelsohn intervention group, the NMES group, and the compound intervention group were compared by using one-way ANOVA for analyzing pre-homogeneity. The results showed that their total scores were significantly different.

### 3.2. Changes in the Swallowing Function, According to Intervention Methods

Differences between the pre- and post-FDS scores of the Mendelsohn intervention group, the NMES group, and the compound intervention group were analyzed by using ANCOVA (Table 4). The results showed that there were significant differences in the change of FDS scores among the groups (*p* < 0.05). The change in FDS scores was the highest for the compound intervention group, followed by the Mendelsohn intervention group and the NMES group, in the descending order. In terms of the sub-domain of FDS, the compound intervention group revealed the highest changes in laryngeal elevation and epiglottic closure and coating of the pharyngeal wall after swallowing fluid, as well.

### 3.3. Changes in Swallowing–Quality of Life According to Intervention Methods

Differences between the pre- and post-SWAL–QOL scores of the Mendelsohn intervention group, the NMES group, and the compound intervention group were analyzed by using ANCOVA (Table 5). The results showed that there were significant differences in the change of SWAL–QOL scores among the groups (*p* < 0.05). The change in SWAL–QOL scores was the highest for the compound intervention group, followed by the Mendelsohn intervention group and the NMES group, in descending order. In terms of the sub-domain of SWAL–QOL, the compound intervention group had the highest changes in symptoms and frequency, communication, and sleep.

## 4. Discussion

This study evaluated the effects of the compound intervention program (Mendelsohn maneuver + NMES) that was conducted for 8 weeks in patients with sub-acute swallowing disorders due to cerebral infarction on the swallowing function. The results showed that the changes in FDS scores and SWAL–QOL score were different among groups: The compound intervention group had the highest FDS scores and SWAL–QOL score, followed by Mendelsohn, and the NMES group had the lowest. Similar to the results of this study, previous studies [25,26] that examined the effects of a swallowing intervention also reported that traditional swallowing-disorder therapies, such as posture change and diet modification, consistently improved the swallowing function. Particularly, it has been found that the Mendelsohn maneuver, which shrinks the fundus of the tongue to its maximum, makes the tongue contact the pharyngeal wall, and closes the airway at the same time by maintaining the highest position of the larynx, is an effective treatment for patients with swallowing disorders in the pharynx stage [10].

On the other hand, the effects of NMES are still controversial. It has been reported that the increased muscular strength owing to the application of NMES to the muscle related to swallowing induces the activation of Type II muscle fiber (fast-twitch muscle fiber), as well as a mechanism similar to the increased muscular strength by voluntary exercise following a high-intensity/low-frequency strength-enhancing protocol [27]. Numerous studies have shown that NMES significantly enhanced the recovery of the swallowing function [28,29]. Li et al. [14] examined the effectiveness of a swallowing therapy in 118 patients for 4 weeks and reported that the combination of NMES and a traditional dysphagia therapy improved the swallowing function than the NMES-only treatment or the traditional rehabilitation therapy only treatment. Bulow et al. [28] compared the effects of traditional swallowing disorder therapies with those of NMES, using a randomized controlled trial (RCT) design and showed that there was no significant difference between them. Contrarily, the retrospective cohort study of Blumenfeld et al. [30] reported that NMES improved the swallowing function significantly more than the traditional therapy. Carnaby-Mann & Crary [13] conducted a meta-study on NMES and analyzed 255 dysphagia patients who satisfied selection criteria. They showed that NMES had an advantage over other treatments. However, the meta-study of Chen et al. [29] reported that there was no significant difference between NMES only treatment and a traditional therapy only treatment.

On the contrary, a number of studies questioned the effectiveness of NMES. Ludlow et al. [16] reported that NMES could rather interrupt the swallowing by stimulating the thyrohyoid muscle and lowering the hyoid bone. The systematic review study of Steele et al. [17] also pointed out that previous studies on NMES posed questions in terms of stimulus location, subject selection, and the accuracy of evaluation methods. Additionally, it was reported that the physiological effects of NMES on the cervical muscle and the swallowing process have not been clearly investigated and it can be applied only to the limited number of patients [15]. In summary, the basis for the application of NMES as an individual therapy is still lacking because NMES only treatment did not show significant improvement in the recovery of the swallowing function or the effects of NMES were not significantly different from those of a traditional swallowing therapy [12].

Nevertheless, it is noteworthy that this study showed that the combination of NMES and Mendelsohn improved the recovery of the swallowing function and the swallowing-related quality of life significantly more than the NMES only treatment or the Mendelsohn only treatment. The result implies that NMES might be effective when it is combined with a traditional swallowing-rehabilitation therapy rather than a single treatment, because the recovery of the swallowing function depends on the complex and simultaneous improvement of various swallowing-related muscles, not the functional enhancement of a single muscle.

A compound intervention means an intervention method that combines or compounds individual treatment methods [31]. A compound intervention program can be used as an effective intervention method for patients with sub-acute swallowing disorders because a synergistic effect can be expected owing to the combination of individual treatments. Moreover, it can be a strategy that can overcome the limitations of domestic and international studies that have evaluated the effectiveness of a single treatment. Nevertheless, since the compound swallowing intervention has been evaluated rarely, various studies should be conducted to examine the effects of the compound swallowing intervention in the future.

The limitations of this study are as follows. First, it is difficult to generalize the results of this study because this study had a limited number of samples, which were non-randomly sampled at medical institutions in Seoul and Incheon. Subsequent studies need to collect samples nationwide, using diverse sampling methods such as a systematic sampling method. Second, this study could not conduct repeated-measure ANOVA, because of a small sampling size and a risk of missing value. Third, this study used a nonequivalent control group pretest–posttest design. The baseline FDS scores of the three groups were significantly different and the composite arbitration group had the highest score. It is possible that the difference in FDS score among the groups at baseline could affect the difference in improvement of post-intervention swallowing function in this study. Therefore, future studies need to employ a randomized experimental design that can control swallowing function at the baseline level. Fourth, it has been reported that the effects of the NMES treatment varied by stimulus intensity and stimulus frequency. Therefore, future studies are needed to evaluate the effects of stimulus intensity and stimulus frequency.

## 5. Conclusions

The results of this study showed that the simultaneous application of the Mendelsohn maneuver and NMES had a significant effect on improving swallowing–quality of life than when Mendelsohn and NMES were applied alone. The result of this study suggests that NMES can be more effective when it is combined with a traditional swallowing rehabilitation therapy rather than a single intervention method because the recovery of the swallowing function depends on the complex and simultaneous improvement of various swallowing-related muscles, not the functional enhancement of a single muscle. RCT studies are needed to examine the effectiveness of a compound swallowing-intervention program using a large sample size.

## Figures and Tables

**Table 1:** Baseline characteristics of subjects, M ± SD.

Variables	Mendelsohn Maneuver (*n* = 15)	NMES (*n* = 13)	Compound Intervention Program (*n* = 15)	*p*
Gender, *n* (%)				0.31
Male	8 (53.3)	7 (53.8)	9 (60.0)	
Female	7 (46.7)	6 (46.2)	6 (40.0)	
Age (years), m ± sd	63.5 ± 5.7	65.1 ± 9.3	65.0 ± 7.3	0.75
Mean monthly household income, *n* (%)				<0.001
>2,000,000 KRW	5 (33.3)	7 (53.8)	7 (46.7)	
2,000,000–3,000,000 KRW	6 (40.0)	6 (46.2)	5 (33.3)	
<3,000,000 KRW	4 (26.7)	0	3 (20.0)	
Longest occupation				<0.001
Manual workers	6 (40.0)	4 (30.8)	7 (46.7)	
Non-manual workers	1 (6.7)	3 (23.1)	2 (13.3)	
Economically inactive population	8 (53.3)	6 (46.1)	6 (40.0)	
Highest level of education				0.56
Graduation below junior high school	12 (80.0)	11 (84.6)	12 (80.0)	
High school graduation or above	3 (20.0)	2 (15.4)	3 (20.0)	
Time since dysphagia (month)	5.1 ± 1.8	5.5 ± 1.5	4.9 ± 1.5	0.81
FDS *	34.1 ± 21.5	36.7 ± 17.9	31.9 ± 19.8	0.75

* FDS = Functional Dysphagia Scale. NMES = neuromuscular electrical stimulation. KRW= the national currency of South Korea.

**Table 2:** FDS characteristics of the Mendelsohn intervention group, the NMES group, and the compound intervention group at the baseline, Mean ± SD.

FDS	Mendelsohn Maneuver (*n* = 15)	NMES (*n* = 13)	Compound Intervention Program (*n* = 15)	*p*
Total Score	34.1 ± 21.5	37.8 ± 17.9	51.9 ± 19.8	<0.001
LC	1.3 ± 2.3	0.6 ± 1.2	1.3 ± 2.0	<0.001
BF	1.7 ± 2.5	1.0 ± 1.5	1.3 ± 2.1	0.532
ROC	1.3 ± 1.7	1.5 ± 1.5	1.7 ± 1.6	0.085
OTT	1.7 ± 2.2	1.4 ± 2.3	2.1 ± 2.5	0.323
TPS	5.1 ± 5.0	6.5 ± 4.7	7.7 ± 4.3	0.256
LEEC	6.0 ± 6.1	6.2 ± 6.0	7.6 ± 4.6	0.018
NP	1.2 ± 3.4	0.8 ± 2.7	0.9 ± 3.0	0.563
RV	5.8 ± 4.6	5.8 ± 3.1	8.2 ± 3.2	0.001
RPS	4.6 ± 4.2	4.7 ± 3.5	7.6 ± 3.5	0.017
CPWSF	4.5 ± 4.7	6.7 ± 4.5	8.3 ± 3.6	0.001
PTT	1.1 ± 1.7	1.5 ± 1.9	1.4 ± 1.7	0.256

LC: Lip Closure, BF: Bolus Formation, ROC: Residue in Oral Cavity, OTT: Oral Transit Time, TPSF: Triggering of Pharyngeal Swallow Fluid, LEEC: Laryngeal Elevation and Epiglottic Closure, RV: Residue in Valleculae, RPS: Residue in Pyriform Sinuses, CPWSF: Coating of Pharyngeal Wall after Swallow Fluid, PTT: Pharyngeal Transit Time.

**Table 3:** SWAL–QOL characteristics of the Mendelsohn intervention group, the NMES group, and the compound intervention group at the baseline, Mean ± SD.

SWAL–QOL	Mendelsohn Maneuver (*n* = 15)	NMES (*n* = 13)	Compound Intervention Program (*n* = 15)	*p*
Total score	120.8 ± 20.5	111.2 ± 22.1	133.8 ± 18.7	<0.001
Burden	5.3 ± 1.3	6.5 ± 1.5	8.8 ± 1.2	<0.001
Fear	10.9 ± 2.2	8.0 ± 2.0	9.5 ± 2.2	0.085
Eating duration	6.3 ± 1.5	7.0 ± 2.1	9.1 ± 1.3	0.015
Eating desire	10.7 ± 2.2	9.3 ± 2.1	11.2 ± 2.0	0.530
Symptoms and frequency	30.1 ± 7.7	25.1 ± 8.3	33.5 ± 7.2	<0.001
Food selection	7.8 ± 1.4	7.3 ± 1.7	8.3 ± 1.5	0.751
Communication	7.7 ± 1.5	8.1 ± 1.8	7.0 ± 1.8	0.275
Social	11.9 ± 4.1	12.1 ± 4.5	12.3 ± 4.1	0.380
Fatigue	9.2 ± 2.6	8.7 ± 2.1	9.7 ± 2.0	0.581
Sleep	6.0 ± 1.8	6.5 ± 1.7	6.8 ± 2.0	0.070
Mental health	15.5 ± 2.3	11.9 ± 3.3	18.5 ± 2.3	<0.001

**Table 4:** Changes in the pre- and post-FDS scores of the Mendelsohn intervention group, the NMES group, and the compound intervention group, Mean ± SD.

FDS	Mendelsohn Maneuver (*n* = 15)		NMES (*n* = 13)		Compound Intervention Program (*n* = 15)		*p*	Scheffe Test
	Pre	Post	Pre	Post	Pre	Post		
Total Score	34.1 ± 21.5	24.7 ± 20.2	37.8 ± 17.9	32.6 ± 16.2	51.9 ± 19.8	37.5 ± 17.7	0.001	b < a < c
LC	1.3 ± 2.3	0.9 ± 1.8	0.6 ± 1.2	0.4 ± 1.1	1.3 ± 2.0	0.8 ± 1.8	0.371	
BF	1.7 ± 2.5	0.9 ± 1.8	1.0 ± 1.5	0.7 ± 1.3	1.3 ± 2.1	0.8 ± 2.0	0.570	
ROC	1.3 ± 1.7	1.2 ± 1.6	1.5 ± 1.5	1.2 ± 1.6	1.7 ± 1.6	1.3 ± 1.5	0.551	
OTT	1.7 ± 2.2	0.8 ± 2.0	1.4 ± 2.3	1.0 ± 2.1	2.1 ± 2.5	1.6 ± 2.3	0.487	
TPS	5.1 ± 5.0	4.5 ± 4.3	6.5 ± 4.7	6.0 ± 3.8	7.7 ± 4.3	7.0 ± 4.1	0.153	
LEEC	6.0 ± 6.1	3.5 ± 5.5	6.2 ± 6.0	5.1 ± 5.6	7.6 ± 4.6	3.3 ± 4.4	<0.001	b < a < c
NP	1.2 ± 3.4	0.4 ± 1.3	0.8 ± 2.7	0.6 ± 2.0	0.9 ± 3.0	0.6 ± 2.9	0.315	
RV	5.8 ± 4.6	5.2 ± 4.1	5.8 ± 3.1	5.0 ± 3.3	8.2 ± 3.2	7.7 ± 3.1	0.870	
RPS	4.6 ± 4.2	4.3 ± 3.5	4.7 ± 3.5	3.7 ± 3.7	7.6 ± 3.5	7.0 ± 3.3	0.461	
CPWSF	4.5 ± 4.7	2.8 ± 4.3	6.7 ± 4.5	4.3 ± 4.2	8.3 ± 3.6	4.1 ± 3.3	<0.001	a = b < c
PTT	1.1 ± 1.7	0.7 ± 1.6	1.5 ± 1.9	0.8 ± 1.8	1.4 ± 1.7	0.5 ± 1.7	0.385	

Scheffe’s multiple comparison test: a = Mendelsohn maneuver, b = NMES, c = compound intervention program, LC: Lip Closure, BF: Bolus Formation, ROC: Residue in Oral Cavity, OTT: Oral Transit Time, TPSF: Triggering of Pharyngeal Swallow Fluid, LEEC: Laryngeal Elevation and Epiglottic Closure, RV: Residue in Valleculae, RPS: Residue in Pyriform Sinuses, CPWSF: Coating of Pharyngeal Wall after Swallow Fluid, PTT: Pharyngeal Transit Time.

**Table 5:** Changes in SWAL–QOL according to intervention methods, Mean ± SD.

SWAL–QOL	Test	Mendelsohn Maneuver (*n* = 15)	NMES (*n* = 13)	Compound Intervention Program (*n* = 15)	*p*	Scheffe Test
Total score	Pre	120.8 ± 20.5	111.2 ± 22.1	133.8 ± 18.7	0.001	b < a < c
	Post	134.8 ± 21.7	120.5 ± 23.0	152.3 ± 20.1		
Burden	Pre	5.3 ± 1.3	6.5 ± 1.5	8.8 ± 1.2	0.795	
	Post	5.9 ± 1.4	6.7 ± 1.5	9.3 ± 1.2		
Fear	Pre	10.9 ± 2.2	8.0 ± 2.0	9.5 ± 2.2	0.271	
	Post	11.6 ± 2.1	8.5 ± 2.0	10.4 ± 2.1		
Eating duration	Pre	6.3 ± 1.5	7.0 ± 2.1	9.1 ± 1.3	0.381	
	Post	7.7 ± 1.6	8.3 ± 2.0	10.0 ± 1.4		
Eating desire	Pre	10.7 ± 2.2	9.3 ± 2.1	11.2 ± 2.0	0.450	
	Post	11.3 ± 2.3	10.2 ± 2.0	11.8 ± 2.0		
Symptoms and frequency	Pre	30.1 ± 7.7	25.1 ± 8.3	33.5 ± 7.2	<0.001	b = a < c
	Post	34.3 ± 7.4	27.5 ± 8.1	41.8 ± 6.9		
Food selection	Pre	7.8 ± 1.4	7.3 ± 1.7	8.3 ± 1.5	0.275	
	Post	8.5 ± 1.5	8.0 ± 1.8	8.9 ± 1.5		
Communication	Pre	7.7 ± 1.5	8.1 ± 1.8	7.0 ± 1.8	0.015	b < a < c
	Post	8.9 ± 1.3	8.8 ± 1.8	9.1 ± 1.6		
Social	Pre	11.9 ± 4.1	12.1 ± 4.5	12.3 ± 4.1	0.883	
	Post	12.5 ± 4.0	12.8 ± 4.5	12.9 ± 4.0		
Fatigue	Pre	9.2 ± 2.6	8.7 ± 2.1	9.7 ± 2.0	0.570	
	Post	10.5 ± 2.3	9.9 ± 1.9	10.8 ± 1.9		
Sleep	Pre	6.0 ± 1.8	6.5 ± 1.7	6.8 ± 2.0	0.001	b < a < c
	Post	6.7 ± 1.8	7.5 ± 1.8	8.5 ± 1.8		
Mental health	Pre	15.5 ± 2.3	11.9 ± 3.3	18.5 ± 2.3	0.550	
	Post	16.3 ± 2.2	12.8 ± 3.1	19.5 ± 2.2		

Scheffe’s multiple comparison test: a = Mendelsohn maneuver, b = NMES, c = compound intervention program.
